# Detection of critical antibiotic resistance genes through routine microbiome surveillance

**DOI:** 10.1371/journal.pone.0213280

**Published:** 2019-03-14

**Authors:** Zachary M. Burcham, Carl J. Schmidt, Jennifer L. Pechal, Christopher P. Brooks, Jason W. Rosch, M. Eric Benbow, Heather R. Jordan

**Affiliations:** 1 Department of Biological Sciences, Mississippi State University, Starkville, MS, United States of America; 2 Department of Pathology, University of Michigan, Ann Arbor, MI, United States of America; 3 Department of Entomology, Michigan State University, East Lansing, MI, United States of America; 4 Department of Infectious Disease, St. Jude Children’s Research Hospital, Memphis, TN, United States of America; 5 Department of Osteopathic Medical Specialties, Michigan State University, East Lansing, MI, United States of America; University of Illinois at Urbana-Champaign, UNITED STATES

## Abstract

Population-based public health data on antibiotic resistance gene carriage is poorly surveyed. Research of the human microbiome as an antibiotic resistance reservoir has primarily focused on gut associated microbial communities, but data have shown more widespread microbial colonization across organs than originally believed, with organs previously considered as sterile being colonized. Our study demonstrates the utility of postmortem microbiome sampling during routine autopsy as a method to survey antibiotic resistance carriage in a general population. Postmortem microbial sampling detected pathogens of public health concern including genes for multidrug efflux pumps, carbapenem, methicillin, vancomycin, and polymixin resistances. Results suggest that postmortem assessments of host-associated microbial communities are useful in acquiring community specific data while reducing selective-participant biases.

## Introduction

Antibiotic resistance (AR) mechanisms are creating an enormous clinical and financial burden on healthcare systems worldwide, and have greatly contributed to newly emerging pathogens, epidemics, and pandemics [1–3]. In the US, the CDC reports that at least 2 million people become infected with antibiotic resistant bacteria each year, and at least 23,000 people die as a result of those infections [4]. Furthermore, a WHO report issued in May 2014 estimated a yearly cost of $21 to $34 billion attributed to AR within the US healthcare system alone, with 8 million additional days spent in the hospital [5].

United Nations leaders recently committed to global action plans to understand the full scale of AR and increase surveillance to rapidly respond to threats [6]. A significant problem in achieving these goals is the availability of comprehensive, reliable surveillance tools, as most data are collected in association with hospital stays or following antibiotic failure, leaving the majority of data hospital-acquired, and community-acquired resistance underreported [5]. Currently, the US surveils AR bacteria from ill people (CDC), retail meats (FDA), and livestock (USDA) [7]. However, the current method of reporting standards fails to address indigenous bacterial populations residing within natural, commensal microbial communities, and asymptomatic infections of humans that contain antibiotic resistance genes (ARGs) [4, 5, 8]. Therefore, reliable, widespread surveillance methodologies that include both hospital and community populations will be important for discovering and preparing for potential AR threats.

The acquisition and transfer of superbugs such as *Staphylococcus aureus*, *Neisseria gonorrhea*, *Clostridium difficile*, *Klebsiella*, and *Enterobacter*, have arisen through both hospital and community exposure [4], proliferating more-so during civil unrest, violence, famine, natural disasters, and poor or nonexistent hospital or hygiene practices [1]. Increasing concerns of these multidrug resistant (MDR) bacteria across the human population has led to expanded research, leading to the CDC establishing the Antibiotic Resistance Laboratory Network in 2016 to rapidly detect and respond to AR threats from community and healthcare sources (https://www.cdc.gov/drugresistance/solutions-initiative/ar-lab-networks.html).

A CDC study published in April 2018 investigated infection data from the National Healthcare Safety Network from 2006–2015 and discovered that carbapenem-resistant Enterobacteriaceae infections had decreased over the studied timeframe, suggesting increased detection and early response to emerging AR threats have the potential to slow dissemination [9]. Moreover, asymptomatic carriage was detected in 11% of screening tests for carbapenemases in healthcare contacts [10]. These findings underscore the importance of continued surveillance for asymptomatic colonization of antibiotic resistant bacteria, as increased prevalence of these organisms could lead to rapid transmission across human communities, which could result in higher morbidity. Furthermore, antibiotic resistant bacteria asymptomatically colonizing the human microbiome can evolve more recalcitrant pathogens by acquiring mutations with increased transmissibility and dissemination across human populations, due to a lack of detection. For example, in March 2018, the Public Health England Reference Laboratory reported the first case of high-level azithromycin and ceftriaxone resistant *Neisseria gonorrhea* [11]. This discovery of multi-drug resistant *N*. *gonorrhea* raises great concern as it is contracted sexually, difficult to treat, and can asymptomatically colonize humans, thus facilitating global AR dispersal through unaware transmission.

The human microbiome is presumably one of the most accessible and underutilized ARG reservoirs due to the high density of microorganisms [12]. The composition of the human microbiome varies spatiotemporally across anatomical areas both internally and externally [13, 14]. Hence, creating a dynamic and mobile environment of ARG transfer amongst populations. Research of the human microbiome as an AR reservoir, known as the human resistome, has primarily focused on gut associated microbial communities, but data have shown more widespread microbial colonization across organs than originally believed, with organs previously considered as sterile being colonized [15, 16]. Moreover, the characterization of microbial communities carrying AR, collected from healthy human organs, has been largely underexplored [17]. But, in order to determine the extent of resistome dissemination within the human population, it will be necessary to account for the entire microbial ecosystem within the human landscape.

Our study utilized postmortem microbiome sampling during routine autopsy as a method to broadly survey resistomes in a general population. Autopsies are an integral part of routine death investigation, used for determining cause of death, pathology of disease, and surgical treatment success or failure [18]. Along with individual cases, autopsies are beneficial for monitoring the effect of disease outbreaks on public health and are proven, valuable procedures for human anatomy education [19–22]. Recently, Pechal et al. (2018) analyzed the microbial communities from human swab samples collected during autopsy in an industrial-urban population. They found niche differentiation based on anatomical location, community stability up to 48 hours postmortem, and biodiversity correlations with antemortem health conditions [22]. These findings demonstrate the potential value of postmortem microbial sampling from autopsy. The expanded range and availability of autopsy cases can provide sample sizes needed for more realistic cross-sectional data that may include underreported populations, such as asymptomatic carriers. Cases can be studied from a broad range of medical care contact, bridging the gap between hospital- and community-acquired data to provide direct and predictive information towards the presence of ARGs in the community as a whole. Medical examiners may also obtain medical records from autopsy cases that can be used to document past antibiotic treatment regimens and exposures. Additionally, death is universal regardless of age, race, financial status, or ability to obtain medical attention. This allows the reduction of selection- and volunteer-biases in comparison with traditional antemortem resistome surveillance, as study inclusion utilizing the postmortem population presumably has fewer biases.

Microbial samples collected during routine autopsy, therefore, provided a unique opportunity to comprehensively characterize human resistomes by allowing access to anatomical locations not easily accessible antemortem for more in-depth microbiome sampling. Our approach is innovative and timely by expanding the routine bacterial sampling procedures already performed during autopsy and for forensic investigations, in an easily incorporated manner, to create surrogate models of a living human population for biomonitoring ARG presence and abundance associated with clinically important drugs. This method aims to reduce biases in traditional cohorts or from studies of single organisms, and expand current resistome surveillance of entire microbial ecosystems.

## Results

### Bacterial community diversity

**Fig 1:**
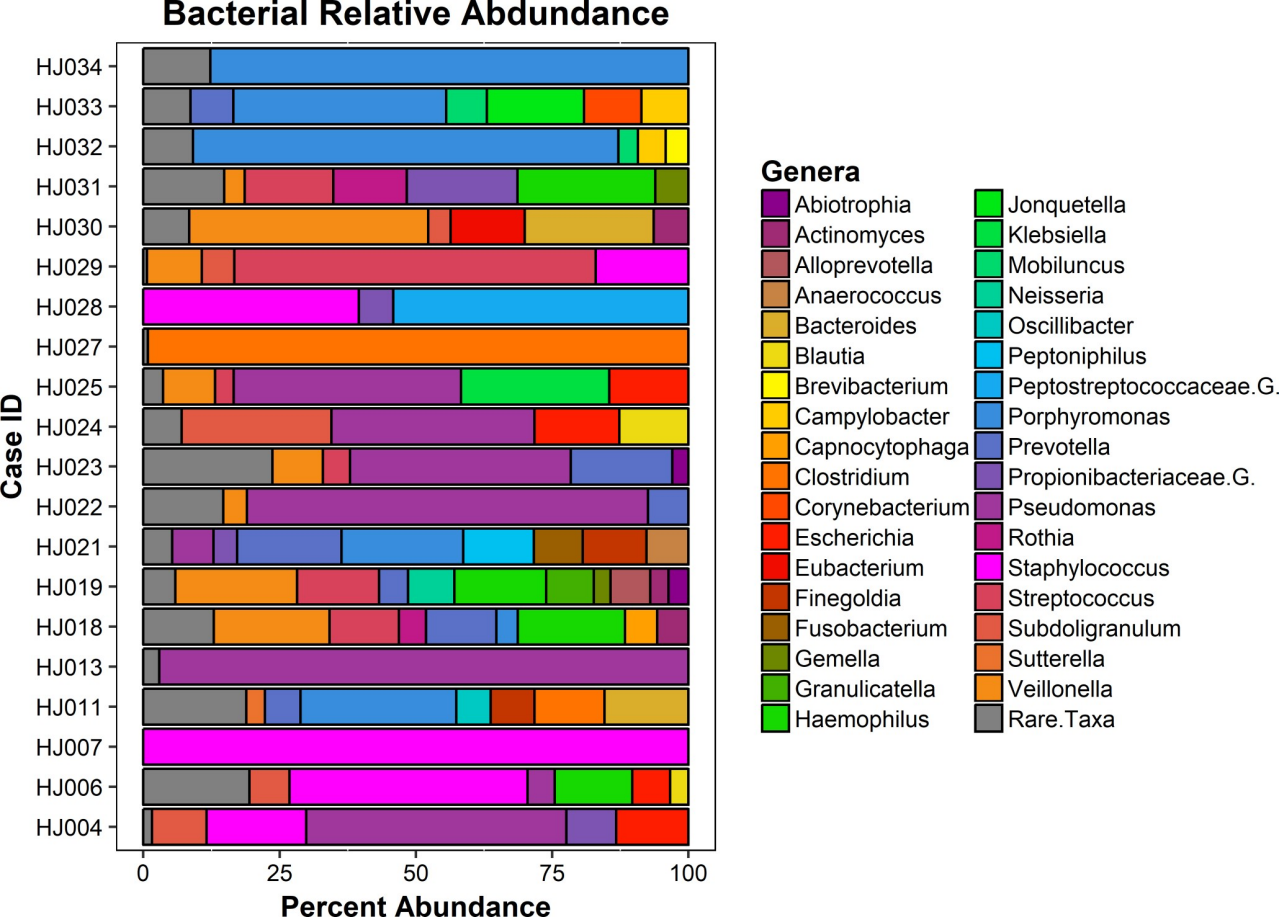
Metagenomic bacterial genera community relative abundance. The relative genera taxonomic level abundance of bacterial communities detected in each case. The highest detected genera in terms of total percentages were *Pseudomonas* (17.7%), *Porphyromonas* (13.4%), and *Staphylococcus* (11.5%). Genera that constituted less than 3% of sample were grouped as rare taxa to reduce sampling noise. Relative abundances were determined using MetaPhlAn v2.0.

Of the 34 cases sequenced, 20 (59%) harbored bacterial metagenomes of sufficient size for ARG detection (S1 Table). Fifty-six unique genera were detected across included cases from metagenome analyses (min = 1, max = 31, mean = 13.95, and SD = 8.97, S2 Table). The top three genera detected (Fig 1) by total percentage were *Pseudomonas* (17.7%), *Porphyromonas* (13.4%), and *Staphylococcus* (11.5%). These genera are consistent with microbiota found in human skin, mouth, and mucosal membranes [14, 23]. Other notable and medically relevant organisms were *Streptococcus* (6.1%), *Clostridium* (5.7%), and *Neisseria* (0.6%), but at lower abundances. Shannon diversity indices of the bacterial communities were tested against the metadata factors (e.g. sex, age, etc.) using ANOVA/MANOVAs, but yielded no significant differences in genera diversity. The similarity in diversity is likely due to the convenient, cross-sectional sampling design, leading to a low sampling size of each metadata group, which was confirmed by statistical power analyses for ANOVA. The similarity of the bacterial communities among cases was relatively high (median Bray-Curtis = 0.7257), with differences in mean community similarity being largely explained by bacterial richness (*r*^*2*^ = 0.62, *P* = 2.5*e*^-5^). To our knowledge, this is the first instance of microbial community data from the human head space so we were not able to compare the results with previous studies.

### Theoretical models for ARGs

**Fig 2:**
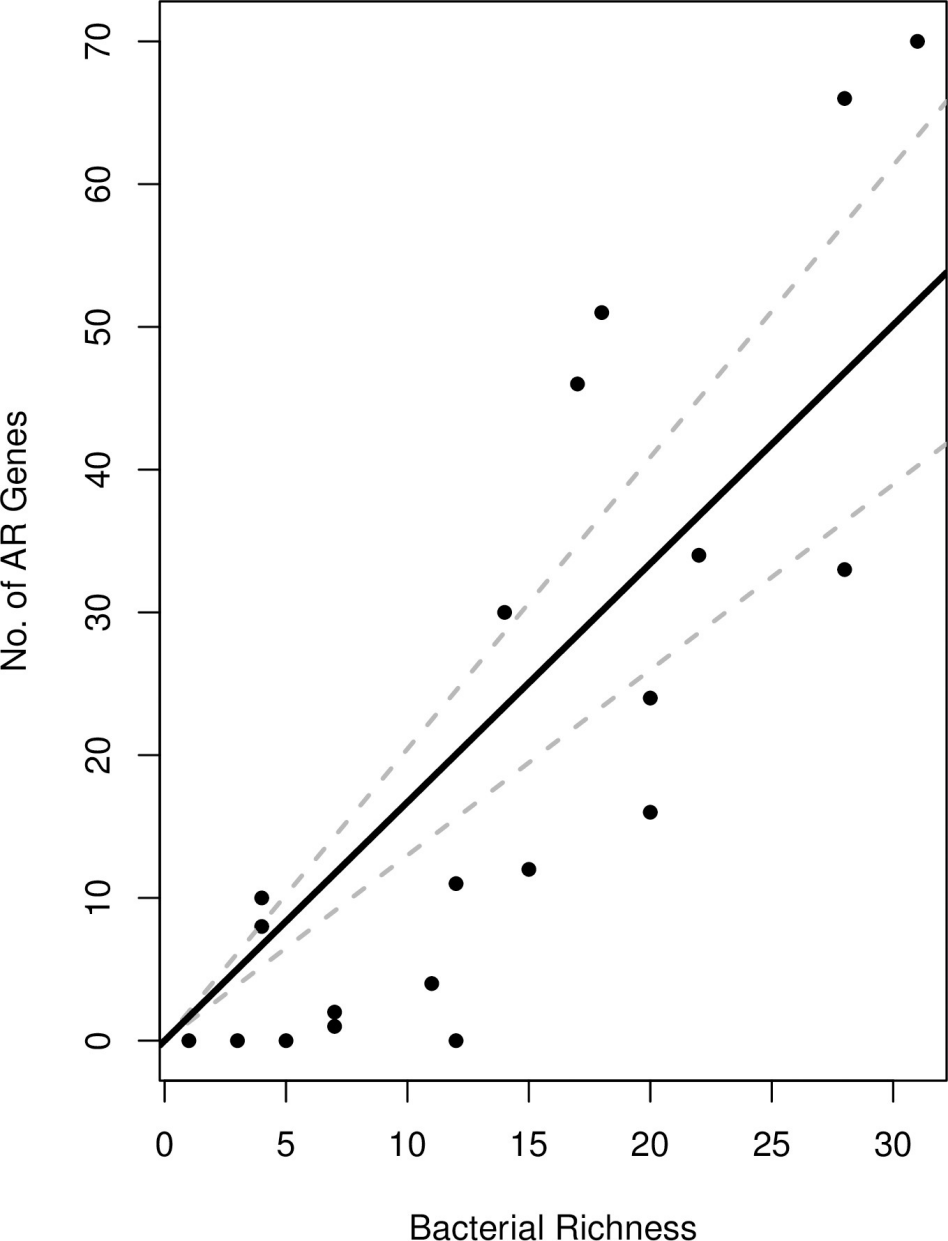
Linear relationship of the metagenomic ARGs as a function of bacterial richness. The dotted slope estimate based on the theoretical model suggests a linear relationship between bacterial richness and the number of resistance genes in the community with a zero intercept and a slope equal to *pN* = 7.66*e*−04 · 2122 = 1.63. The solid slope estimate from a linear regression on the empirical data estimates the slope is 1.67.

The best model was based on the hypothesis that bacterial genera abundance (richness) in these communities is governed by a negative binomial distribution (μ = 13.95, k = 2.45) and that the number of ARGs per taxon is binomially-distributed (*p*_*g*_ = 7.5*e*^-4^). Our estimate of *p*_*g*_ was used to predict the slope of the relationship between taxonomic richness and the number of ARGs as *Np*_*g*_ = 1.59 (95% *CI*: 1.12–2.22) (Fig 2). Using our theoretical model, the relationship with richness accounted for 62% of the variation in the number of resistance genes which suggests a linear relationship between bacterial genera richness and the number of resistance genes in the community with a zero intercept and a slope equal to *pN* = 1.63. The slope estimate from a linear regression on the empirical data estimates the slope is 1.67. While our empirical model was limited by sample size, the theoretical model was not, and yielded a similar slope. This suggests our empirical was enough to detect the linear relationship between genera richness and number of resistance genes. Metagenome size, above the cutoff, was shown not to be a predictor for the number of ARGs detected with a linear regression model (adjusted *r*^*2*^ = -0.03, median = -0.47, IQR = 5.05, *P* = 0.54) and Spearman’s rank correlation (*r*_*s*_ = 0.08, *P* = 0.70).

### Metagenome alignment to ARG database

**Fig 3:**
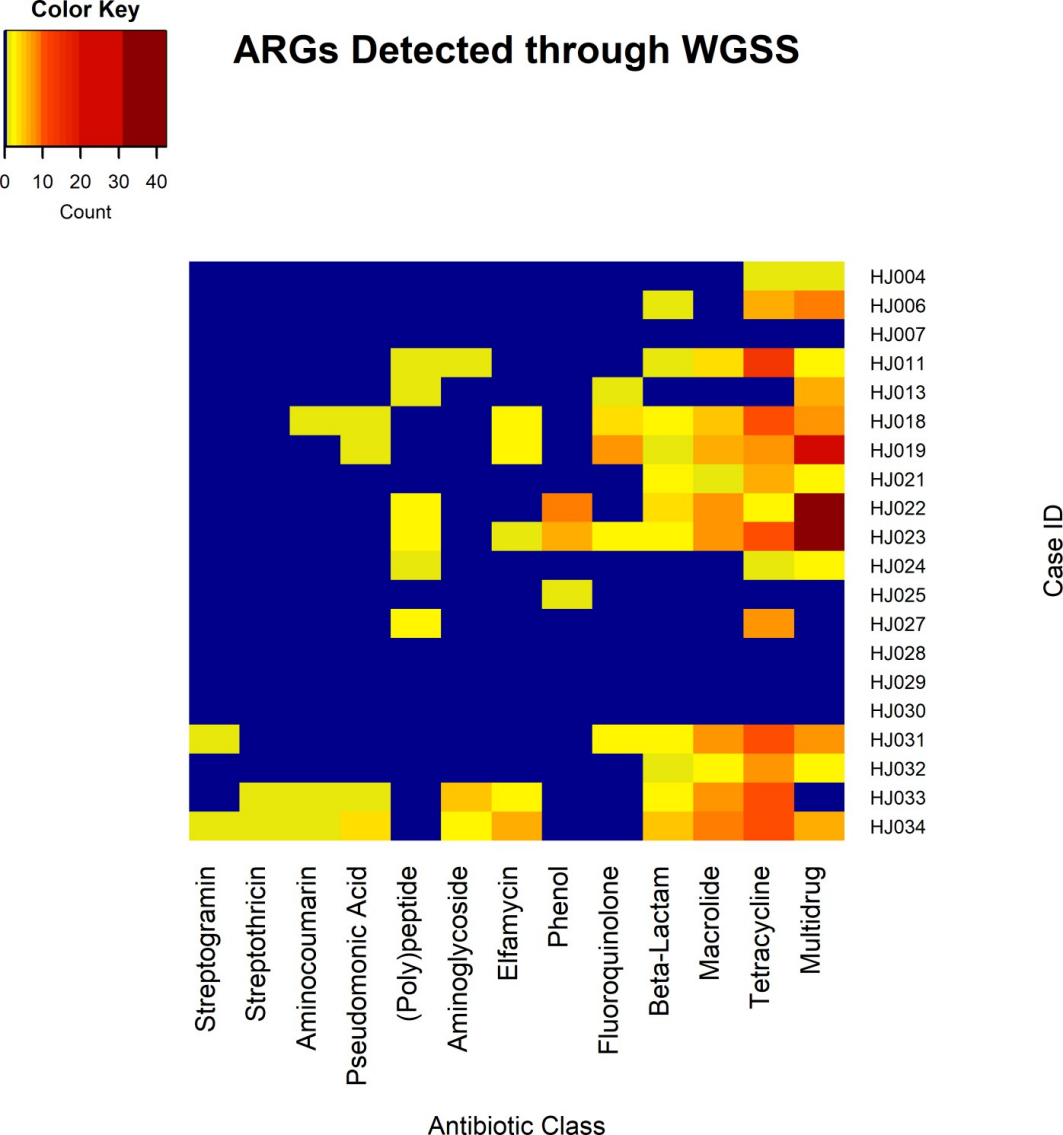
Heatmap visualization of ARGs identified from each case’s metagenome. Resistance genes to streptogramins, streptothricins, aminocoumarins, pseudomonic acids, (poly)peptides, aminoglycosides, elfamycins, phenols, fluoroquinolones, beta-lactams, macrolides, tetracyclines, and multidrug efflux pumps were identified. The color and numbers represent the range of genes found to inhibit antibiotic classes. Blue indicates no detection and red the highest detection.

Ninety-five unique ARGs were found within this survey; however, these genes were detected multiple times for a total of 418 gene detections across the population (min = 0, max = 70, mean = 20.9, median = 11.5 genes, and IQR = 31.5) (Table 1, S1 and S3 Tables). The 95 unique genes created products that either interact with 12 antibiotic drug classes [fluoroquinolone, aminoglycoside, tetracycline, beta-lactam, macrolide, phenol, elfamycin, pseudomonic acid, aminocoumarin, streptothricin, streptogramin, and (poly)peptide], or are part of multidrug efflux pumps. In total, multidrug efflux pump related genes were detected 149 times, tetracycline detected 106 times, macrolide 57, beta-lactam 25, fluoroquinolone 18, phenol 16, elfamycin 15, aminoglycoside 9, (poly)peptide 9, pseudomonic acid 7, aminocoumarin 3, streptothricin 2, and streptogramin 2 (Fig 3). Although in small amounts, we detected genes playing a role in resistance to methicillin and polymixins, including *mec*R1 (2 detected), *arn*A (2 detected), and *pmr*A (3 detected), respectively.

**Table 1:** Table showing identified ARGs among cases using whole genome shotgun sequencing (WGSS) and qPCR assays.

Case	WGSS Genes Present	qPCR Genes Present
**HJ004**	mdtE, tet38	Not Tested
**HJ006**	acrB, acrS, arlR, arlS, blaZ, mdtL, mepA, mepR, msbA, sav1866, tet38, tetQ	Not Tested
**HJ007**	No Detection	Not Tested
**HJ008**	Not Tested	aadA1, SHV, DHA, qnr-23 Group, ermB, mefA, tetB, vanB
**HJ011**	aadA, acrF, arnA, cfxA, ermF, ermX, mdtE, mdtO, tetM, tetO, tetW	Not Tested
**HJ012**	Not Tested	SHV(156G), SHV(238G240E), ermB, ermC, mefA, msrA, mecA
**HJ013**	acrB, arnA, mexB, mexE, mexF, mexK, muxC, oqxB	Not Tested
**HJ015**	Not Tested	IMI/NMC-A, SHV(156G), SHV(238S240K), ACT5/7 Group, ermB, ermC, mefA, msrA
**HJ018**	cfxA, EF-Tu, ermF, hmrM, ileS, mefA, mel, mtrD, parY, patB,	msrA
	pmrA, rlmA(II), TEM, tetA46, tetA60, tetB46, tetB60, tetM, tetQ	
**HJ019**	cfxA, EF-Tu, ermB, ermF, farA, farB, hmrM, ileS, macA, macB,	SFO-1, SHV(238S240K), ermB, ermC, mefA, msrA
	mefA, mel, mtrD, mtrR, patB, pmrA, rlmA(II), tetA60, tetB60, tetM	
**HJ020**	Not Tested	SHV, SHV(156G), SHV(238G240E), MOX, ermB, ermC, mefA, msrA, tetA, tetB, mecA
**HJ021**	cfxA, ermF, lsaC, tet32, tetM, tetQ	ermB, ermC, mefA, msrA, tetA
**HJ022**	acrB, arnA, cfxA, cpxR, ermF, ermX, mefA, mel, mexA, mexB, mexC,	SHV(238S240K), ermB, mefA, msrA, mecA
	mexD, mexE, mexF, mexI, mexK, mexN, mexP, mexQ, mexW, mexY/amrB,	
	mexY/amrB, muxB, opmH, oprM, PDC, pmpM, smeE, tetQ, tetW, triA, triC	
**HJ023**	acrD, arnA, cfxA, cpxR, EF-Tu, ermB, ermF, ermX, lsaC, mefA, mel, mexB,	SHV(156G), ermB, ermC, mefA, msrA, vanB, mecA
	mexC, mexD, mexE, mexF, mexI, mexK, mexN, mexP, mexQ, mexW, mexY/amrB,	
	muxB, opmH, oprM, patB, pmpM, rlmA(II), smeB, tetA60, tetM, tetO, tetW, triA, triC	
**HJ024**	acrB, acrF, pmrE, tetW	SHV(156G), SHV(238S240K), ACT 5/7 Group, MOX, OXA-50 Group,
		OXA-51 Group, ermB, ermC, mefA, msrA, vanB, mecA
**HJ025**	cpxR	SHV, SHV(156G), SHV(238G240E), MOX, OXA-50 Group, ermB, mefA, oprJ, oprM
**HJ026**	Not Tested	SHV, SHV(156G), SHV(238G240E), MOX
**HJ027**	mprF, tetA(P), tetB(P)	SHV, SHV(156G), SHV(238G240E), MOX, ermB, ermC, mefA, msrA, mecA
**HJ028**	No Detection	ermB, ermC, mefA, msrA, mecA
**HJ029**	No Detection	SHV, SHV(156G), SHV(238G240E), MOX,
		ermB, ermC, mefA, msrA, tetA, tetB, vanB, mecA
**HJ030**	No Detection	SHV, SHV(156G), SHV(238G240E), ermB, ermC, mefA, msrA, tetB
**HJ031**	blaZ, cfxA, ermB, hmrM, mefA, mel, patB, pmrA,	Not Tested
	rlmA(II), TEM, tetK, tetB46, tetM, tetO, tetW, tetX, vgaA	
**HJ032**	ermF, lsaC, TEM, tetM, tetQ, tetT, tetW	Not Tested
**HJ033**	aad(6), ant(6)-Ia, aph(3')-Ia, aph(3')-IIIa, cfxA, EF-Tu, ermC,	Not Tested
	ermF, ermG, ermX, ileS, mecR1, parY, sat-4, tetM, tetO, tetQ, tetW	
**HJ034**	aad(6), ant(6)-Ia, aph(3')-IIIa, arlS, blaB, blaZ, cfxA, EF-Tu, ermA,	Not Tested
	ermC, ermF, ermX, ileS, lsaC, mecR1, mefA, mphC, mtrA, norA, parY,	
	qacA, qacA/qacB, sat-4, tetK, tetM, tetO, tetQ, tetW, tetX, vgaA	
**HJ035**	Not Tested	ermB, mefA, msrA, mecA
**HJ036**	Not Tested	aac(6)-Ib-cr, aadA1, ermB, ermC, mefA, msrA, mecA
**HJ037**	Not Tested	aadA1, DHA, OXA-51 Group, ermB, mefA, msrA, tetB
**HJ038**	Not Tested	ermB, mefA, msrA, tetB, mecA

### Quantitative PCR array for ARGs

**Fig 4:**
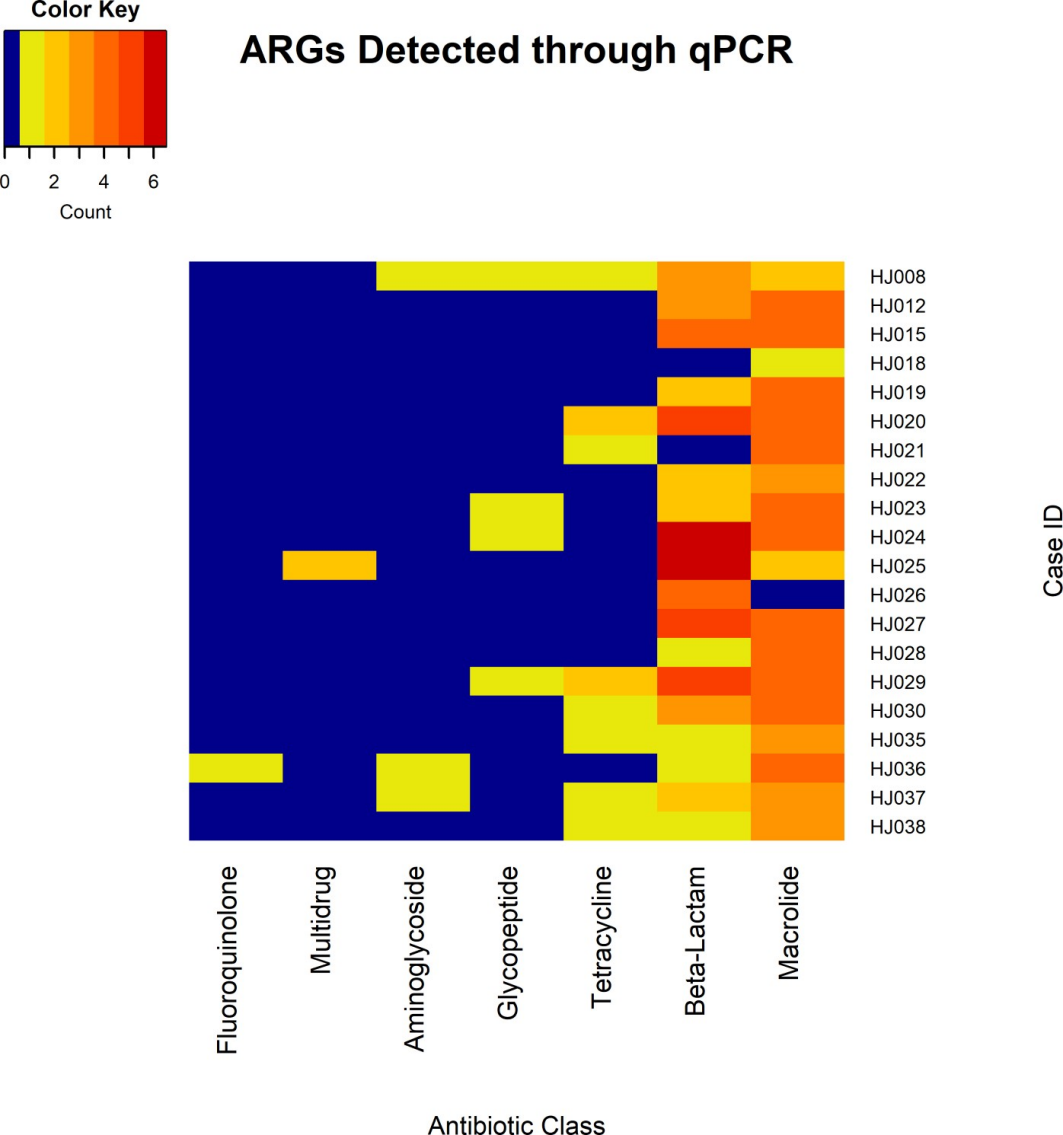
Heatmap visualization of genes related to particular antibiotic classes found in each case through using ARG qPCR arrays. Resistance genes to fluoroquinolones, multidrug efflux pumps, aminoglycosides, glycopeptides, tetracyclines, beta-lactams, and macrolides were discovered. Color and numbers represent the range of abundance of genes found to inhibit antibiotic classes. Blue represents no detection and red the highest detection.

In total, 24 unique ARGs were detected across qPCR analyzed cases, of which 18 were detected multiple times leading to a total of 141 gene detections within the study population (min = 1 gene, max = 12, mean = 7.05, and SD = 2.70) (S1 and S3 Tables). These genes were found to be associated with resistance to 6 antibiotic drug classes (fluoroquinolones, aminoglycosides, glycopeptides, tetracyclines, beta-lactams, and macrolides), or part of multidrug efflux pumps. In total, genes expressing resistance to macrolides were detected 65 times, beta-lactams 56 times, tetracycline 10 times, glycopeptide 4 times, aminoglycoside 3 times, multidrug efflux pumps twice, and fluoroquinolones once (Fig 4). Genes detected in the highest abundance were *erm*B and *me*fA. Notably, genes were detected that play roles in resistance to carbapenems, vancomycin, and methicillin, including OXA groups (4 detected), *van*B (4 detected), and *mec*A (11 detected), respectively, across all cases.

## Discussion

All but one case yielded detection of ARGs associated with activity against multiple antibiotic classes. Macrolide resistance genes were most common in qPCR assays, while multidrug efflux pumps were common in metagenomes. However, both tetracycline and beta-lactam resistance genes were widely detected. Additionally, we detected clinically relevant ARGs associated with polymixins, carbapenem, vancomycin, and methicillin resistance. Differences between the two methods is likely due to the specific targeting of 84 genes in the qPCR assay while the metagenomic sequence alignment detected all genes in the CARD. Additionally, when comparing the two methods, qPCR assays can detect specific genes in lower abundances than sequencing due to the nature of targeted amplicon amplification of qPCR while sequencing can miss low abundance genes. Metagenomic sequencing is beneficial in that it can detect a broad range of genes, and qPCR detection is beneficial for relatively rapid and specific gene detection. Both of these methods can yield valuable surveillance data depending upon whether the research goal is to determine which genes are present in a population (sequencing), or if determining the abundance of a specific array of genes (qPCR) is the aim. However, underreporting of ARGs could occur from both methods. Underreporting can occur with qPCR assays as this method focuses on specific genes and gene detection is often based on semi-quantitative data. Underreporting in sequence data can be limited by sequencing depth and the database being used. It is also important to note that our qPCR analysis focused on DNA pooled from multiple body sites while sequencing analysis focused on the brain space. Therefore, it is not completely clear the extent to which body site affects which ARGs are detected and in what abundance. However, our methodology allowed detection of known ARGs of concern in body sites besides the human gut, which has largely been the resistome study focus.

We also detected genera containing common antibiotic resistant species, such as *Clostridium*, *Pseudomonas*, *Staphylococcus*, and *Neisseria*. While these genera are expected to be present as they are natural commensals of the human microbiota, the presence of the genera along with the presence of their known AR determinants within the same sample are cause for increased concern as the genes needed for antibiotic resistance may be already present in their genome or may be acquired through horizontal gene transfer and natural competence. For example, with HJ024 and HJ025, we detected *Pseudomonas* as well as the OXA-50 carbapenemase gene associated with carbapenem-resistant *P*. *aeruginosa* (ARO:3001796) [24].

Previous human gut resistome metagenomic analyses have shown the human gut contains high amounts of opportunistic pathogens and antibiotic resistance genes belonging to 50 of the 68 known antibiotic classes at an average of 21 per sample [25–27]. For instance, one study investigated resistomes from 162 people across 3 populations and found that genes for multi-drug resistance, tetracycline, and macrolides were among the most abundantly identified [26]. Another recent study analyzed fecal metagenome datasets from 180 healthy individuals across 11 countries, and detected 20 of 24 ARG types in the structured Antibiotic Resistance Gene Database with an average of 21 ARGs identified per sample [28]. However, the latter study found an average of 117 ARGs identified per sample. Our data presented here show ARGs detected at an average of 20.9 ARGs per sample during metagenomic analyses. These differences likely reflect differences in sample size and collection sites, as our samples for metagenomic analyses originated from interhemispheric fissure and trabecular space, rather than fecal samples. Notwithstanding, our results were similar with these studies in that among the ARG classes identified from our sampleset, genes for multi-drug resistance were most abundantly detected, followed by ARGs for tetracycline, macrolides, vancomycin, and bacitracin, among others [28]. These were also found in highest abundance in our work. The concurrence of our data showing similarly detected ARG classes with those from published metagenome-based antemortem resistome studies is not surprising, given the recent finding that postmortem microbiome communities show stability and correlate with antemortem health conditions up to 48 hours postmortem, a time when the majority of our samples were taken [22], and our findings further underscore the potential value of postmortem microbial sampling from autopsy to uncover ARGs from a broad population.

The Infectious Diseases Society of America has referred to the six most life-threatening MDR nosocomial infectious bacteria as the “ESKAPE pathogens” (*Enterococcus faecium*, *S*. *aureus*, *Klebsiella pneumoniae*, *Acinetobacter baumannii*, *P*. *aeruginosa*, and *Enterobacter* species [29]. We detected ARGs associated with all six “ESKAPE pathogens”, including genes that encode products for resistance-nodulation-division (RND) multidrug efflux pumps, which have been shown to provide resistance to carbapenems, fluoroquinolones, chloramphenicol, and aminoglycosides to Gram-negative bacteria [30]. Detecting these genera and associated genes is alarming as it demonstrates potential for genera to either have or acquire resistances. However, the presence of these ARGs does not necessarily correlate to expression and transmission, and functional confirmatory tests should be performed for genes in question.

We detected OXA genes associated with carbapenem resistance in three cases (HJ024, HJ025, and HJ037) and *pmr* genes associated with polymixin resistance in four cases (HJ018, HJ019, HJ024, and HJ031), with one case containing both genes (HJ024). Carbapenems and polymixins are both antibiotics used as a last line of therapy against life-threatening infections, including antimicrobial strains [31, 32]. Carbapenems are the most effective broad spectrum beta-lactam antibiotics used to treat severe infections and are known to be less vulnerable to resistance mechanisms. However, resistance in Enterobacteriaceae associated with carbapenemases has rapidly disseminated across the globe in the past decade. Carbapenem-resistant Enterobacteriaceae (CRE) was originally discovered in a healthcare setting, but since these AR determinants are being detected on mobile genetic elements it is becoming a growing issue that CRE will disseminate through the public and the number of cases will continue to increase [24, 33]. Polymixins, including the antibiotic colistin, are antimicrobial peptides used to treat MDR and have been heavily relied upon to clinically treat carbapenem resistant bacteria. Polymixin resistance has been found to be both intrinsic and acquired, with reports of resistance increasing. Recently, human isolates of *K*. *pneumoniae* and *Escherichia coli* were found to harbor a plasmid-mediated polymixin resistance gene, suggesting horizontal gene transfer had taken place [34]. It is important to note that none of the cases containing resistance determinants for carbapenem or polymixin resistance within our study were hospital-related deaths. While it is possible the cases had contact with a healthcare setting sometime prior to death, the individuals were considered part of the public community at the time of death. Emergence of resistance to these “last line” antibiotics is a tangible public health threat, therefore it is vital to monitor and understand dissemination mechanisms between resistomes at a broad and global scale, and account for ARG reservoirs from both hospital and community settings.

In three separate instances, we also detected both determinants of methicillin and vancomycin resistance from the same case, though not all components of the vancomycin cassette was detected (HJ023, HJ024, and HJ029). Concerns associated with methicillin resistant *S*. *aureus* (MRSA) are well characterized, and vancomycin has been widely used for therapy against MRSA. Unfortunately, vancomycin resistance has spread with unanticipated rapidity and is now encountered by hospitals in most states along with recent reports of vancomycin resistant *S*. *aureus* [35–39] [40–42]. Finding both determinants in a single case suggest genes can be exchanged between organisms, potentially leading to an organism acquiring resistance to both antibiotics.

Many bacterial strains are naturally competent for DNA uptake and have high transformation rates [43, 44], while other strains are rapidly evolving by cooperative mechanisms for antibiotic inactivation [45]. Resistance genes can be disseminated vertically when new generations inherit resistance genes, or horizontally by the sharing or exchange of genetic material between bacterial strains [1, 44, 46]. We investigated the impact of bacterial richness to ARG abundance with a theoretical model relating ARG abundance to the taxonomic richness in postmortem samples to emphasize the importance of antemortem microbial community structure. We found that models accounting for over dispersion in microbial richness described the data better than those accounting for different probabilities of carrying ARGs for different taxa. Therefore, theory predicts that cases with diverse microbial communities are likely to carry increased numbers of ARGs, regardless of taxon identity. These results differ from a recent study showing that ARG subtypes had a significant correlation with microbial community suggesting bacterial phylogeny might shape ARG distribution [28]. Differences in sample size and analysis methodology may account for this variance and further investigation is warranted to draw more concrete conclusions. Notwithstanding, our data imply that our postmortem sampling methodology corroborates the idea that mechanisms of horizontal gene transfer among species has a greater influence than vertical spread within lineages on the number of ARGs detected. The lack of a relationship between metagenome size and number of ARGs detected shows that having a larger metagenome does not predict gene frequency, suggesting that bacterial richness may account for this variance.

We have demonstrated that sampling the human postmortem microbiome through routine autopsy serves as a surrogate model for investigating the human ARG carriage. Previous studies by our group and others have demonstrated human postmortem microbiome stability for up to two days after death, and closely represents antemortem microbial composition; thus our microbial samples from cases, with detected ARGs, are expected to be associated with the living individual (S1 Table)[22]. Our methodologies allowed access to sampling areas that otherwise would not be routinely accessible in the living, and underscored the value of routine autopsy collected samples for a holistic ARG surveillance approach, reducing potential biases of traditional surveillance methods. We recognize that specific microbial community taxa may become enriched or excluded following host death and prior to sampling. In fact, the few cases with PMIs over 48 hours contained high read counts which may correlate to the increase of bacterial growth immediately following death. However, the extent of this enrichment has not been investigated or reported, and it is doubtful that ARGs arise in this short postmortem interval and have a significantly divergent profile from what is present during life, though manners of death, postmortem wounds, or similar contaminating events at the sample site would require further consideration. It is also important to note that detection of a specific ARG does not necessarily translate to the presence or viability of a specific organism, activation of the gene, or ability to exchange the gene to other organisms. Targeted functional analyses will be useful for these determinations. Yet, the presence of an ARG demonstrates resistance and transmission potential. These data are an important first step in determining baseline information for more targeted studies.

While the use of sensitive DNA techniques to discover AR determinants in the population is not novel, we have for the first time demonstrated the application of postmortem microbiome sampling during routine death investigation as a tool for robust antibiotic resistance surveillance. Given the breadth of the global population, our sample size was small and geographically focused in comparison. However, data from our study shows feasibility and value for implementation of such methodology at a larger scale. This method of sampling can potentially reduce biases found in studies and cohorts where living participants are used, as autopsies include a wide-range of demographics that provide a closer snapshot of the population rather than studies based on participant volunteering which might over- and/or underrepresent key demographics. The demographics of participants who volunteer for sampling may not accurately represent the diversity of the population, while autopsy can provide a more accurate sampling population since death is universal across demographics. Medical Examiner’s also have the ability to request medical records of the deceased (if allowable by state law), which can be used to determine if the individual had recent or past treatments, such as antibiotic usage.

We have demonstrated the viability of both qPCR and sequencing detection methods with the applications and limitations of each. We propose metagenomic sequencing can provide a broad analysis of the ARGs present in a geographic location while qPCR assays can be used to monitor specific genes of greatest concern. Currently, whole genome sequencing is likely too costly to implement on a population wide scale, but sequencing cost continue to decrease as sequencing sensitivity increases. Lowering costs may allow AR determinant surveillance sequencing techniques to be executed in areas deemed high risk, for aiding public health officials in knowledge of specific resistome dissemination in a community, and can provide high financial return by predicting and preparing potential outbreaks.

The increased threats of multidrug resistant bacteria has heightened the need for AR carriage surveillance, particularly those colonizing asymptomatically, across communities. However, current reporting methods are predominately healthcare related and fail to address indigenous bacterial populations and asymptomatic infections in humans of both hospital and community populations. Data from our study have shown 1) ARGs associated with bacteria of the greatest public health concern, 2) horizontal gene transfer likely drives AR spread, and 3) the utility of the autopsy as an invaluable tool towards public health surveillance. Our data suggest that postmortem assessments of host-associated microbial communities are useful in acquiring community specific resistome data while reducing volunteer and selective-participant biases. Further, these procedures could provide data from a robust cross-section of populations leading to early identification of ARG dissemination from within the human AR reservoir. The ability to monitor ARG dissemination to such a degree would allow source tracking, outbreak preparation, and treatment alternatives to help reduce AR selective pressure needed by bacteria to maintain resistance. This methodology aims to focus on preventative measures instead of reactive medicine. Therefore, we propose that routine autopsy microbial sampling and metagenomic analysis provides the tools necessary to expand AR surveillance across the human populations. Data from this work demonstrates the ability to detect multiple ARGs associated with known life-threatening bacteria, which could otherwise go undetected as they disperse across the living population.

## Methods

### Wayne county medical examiner information

The Wayne County Medical Examiner receives approximately 3,500 bodies for death investigation, annually. The U.S. Census Bureau July 2016 census details Wayne County, MI, USA as having a population of 1,749,366. Persons under the age of 5 years make up 6.6%, under 18 years make up 24.0%, and equal to or above 65 years make up 14.4% of the population. Females were 51.9% of the population. Race origins of white alone make up 54.6%, black alone make up 39.2%, American Indian or American Alaskan make up 0.5%, Asian alone make up 3.2%, and two or more races make up 2.5% of the population.

### Sample collection and DNA extraction

Microbial communities were collected from thirty-nine cases received to the Wayne County Medical Examiner’s Office, MI as part of routine death investigation, previously described in Pechal et al. (2018)[22]. All swab sampling was performed by trained personnel at the Wayne County Medical Examiner’s Office. Inclusion criteria for cases in this dataset were chosen to reflect demographics of the living human population and included: age 10–80 years old; male or female; black or white. The following information for collected for each case: sex, age, ethnicity, manner of death (natural, accidental, homicide, or suicide), body mass index (BMI) as determined at autopsy by a board certified forensic pathologist. BMI was classified into weight classifications according to World Health Organization recommendations: underweight [<18.5], normal [18.5–24.9], overweight [25–29.9], or obese [30+]. Cases were grouped by age (Y = youth [0-17yo], YA = young adult [18-25yo], A = adult [26-40yo], MA = middle aged adult [41-60yo], OA = old aged adult [61+yo]) based on a modified version of the United States Library of Congress preferred terms for life stages/age groups with the early ages 0–17 years being classified as “youth”. The death event location, or where a body was discovered, were classified broadly as: indoors, outdoors, or hospital (S1 Table).

Samples were collected in conjunction with a previous study. Seven anatomical locations were collected using DNA-Free, sterile cotton-tipped applicators: the external auditory canal, eyes, nose, mouth, umbilicus, rectum, and trabecular space of the occipital bone. Swabs were also taken from the interhemispheric fissure in some cases (S1 Table). For each anatomic location, an individual swab was physically rubbed while rotating the swab for 3–5 second, then the cotton end of the applicator was placed in an individual, sterile microcentrifuge tube filled with 200 μl of molecular grade ethanol. Samples were stored -20°C until further processing. All swab sampling was performed by trained personnel at the Wayne County Medical Examiner’s Office (Detroit, MI). Genomic DNA was extracted from applicator tips, as previously described, following the manufacturer’s instructions for the PureLink Genomic DNA Mini Kit with the following modification: 15 mg/mL of lysozyme was added during the lysis step for reaction [22]. DNA was quantified using the Quant-iT dsDNA HS Assay kit and a Qubit 2.0. DNA elutions were stored at -20°C. DNA from the interhemispheric fissure and trabecular space were utilized for library preparation (described below), and submission for high-throughput sequencing using the Illumina HiSeq 2000 platform. All other anatomical locations were pooled for quantitative PCR analysis as described below.

### Library preparation and whole genome shotgun sequencing (WGSS)

DNA from 34 cases were processed for sequencing, with 26 cases having solely used in this study. DNA from trabecular space DNA and eight cases having DNA from both the trabecular space and interhemispheric fissure were used to create libraries for whole genome shotgun sequencing using the NEBNext Ultra DNA Library Prep Kit for Illumina and NEBNext Multiplex Oligos for Illumina (Dual Index Primers Set 1) according to manufacturer protocols (S1 Table). Briefly, DNA ends were repaired and Illumina adapters were ligated to the newly repaired ends. The adapter-ligated DNA was cleaned without size selection and enriched through PCR to add the universal and index primers allowing for multiplexing. PCR amplicons were cleaned, combined, and stored at -20°C until sent to St. Jude Children’s Research Hospital (Memphis, TN) for whole genome shotgun sequencing. No “blank” negative control libraries were created as these would likely negatively affect sequencing depth and coverage in multiplexed libraries since all our samples were ran on a single sequencing lane. Libraries were sequenced using an Illumina HiSeq 2000 platform creating the 100 bp paired-end sequence reads for each sample that were parsed out along with the removal of the Illumina adapters and primers by the St. Jude Children’s Research Hospital streamlined post-sequencing processing protocol.

### Metagenome assembly and antibiotic resistance gene (ARG) detection

Paired-end sequence reads for each sample were trimmed to remove low quality nucleotides, remaining adapters, or primer sequences using Trimmomatic v0.33. Paired-end reads for each sample were assembled *de novo* into metagenomes using the default parameters and multiple k-mer values using metaSPAdes v3.10.1, with exception to HJ031, which was too large to assemble using multiple k-mers and was assembled using a single k-mer of 21[47]. Sequences from 14 of the 34 cases assayed using metagenomics sequencing were removed from the analysis due to poor sequencing depth and metagenome size, as discussed below (S2 Table). A nucleotide BLAST database was created using ARG FASTA protein homolog sequences found in the Comprehensive Antibiotic Resistance Database (CARD) v1.1.8, updated May 2017[48–51]. Contigs from each metagenome were screened for ARGs using a local BLASTn search with an e-value cutoff of 1.0e-10 to ensure high alignment confidence. A detection limit cut-off was created at the size of the smallest metagenome that provided a positive gene hit (5,268,350 nucleotides). Any metagenomes below the detection limit were considered too small to analyze. When a sequence segment aligned to multiple ARGs, the hit with the lowest e-value, along with the longest alignment length was considered the true match. There were a total of 418 query hits with an average length of 538 bp (min = 47, max = 3184, SD = 550) and average percent identity of 91% (min = 72, max = 100, SD = 9). Resulting data from cases with DNA from both the trabecular space and interhemispheric fissure were combined to represent the detected ARGs.

### Bacterial community analysis

For each case analyzed for metagenome ARG detection, paired-end sequence reads were trimmed to remove low quality nucleotides and remaining Illumina adapter or primer sequences using Trimmomatic v0.33[52]. Bacterial detection in the interhemispheric fissure was possibly limited in some samples by the small amount of DNA recovered from the site, but when DNA was sufficient, diversity analyses closely resembled trabecular space communities of the same case. For this reason, sequence reads for the trabecular space and interhemispheric fissure were combined for each case to create a community profile of the brain space and for overall ARG detection, in order to maintain analyses consistency across cases and analytical methods. Taxonomic profiling and relative abundance of bacteria at the genera level were estimated using MetaPhlAn v2.0[53]. Genera that constituted less than 3% relative abundance of the sample were grouped as rare taxa to reduce sampling noise, though this grouping of rare taxa was not performed during diversity analyses.

### Theoretical models for metagenome ARGs in a microbial community

The empirical data suggested a relationship between the number of taxa (richness) in the postmortem bacterial communities and the number of ARGs found in the metagenomes from each host (Fig 2). We started by hypothesizing that the number of ARGs (*g*_*i*_) in a community (*i*) is the product of the number of taxa in the community (*s*_*i*_), and the expected number of ARGs carried by each *pN* (where *p* is the probability of a bacterial taxon carrying a ARG, and *N* is the number of ARGs that can potentially be detected):

gi=si·pN

This equation was for a straight line with a *y*-intercept of zero (when there are zero species in a community, there should be zero resistance genes), and a slope of *pN*. For the current project, our model indicated up to *N* = 2122 ARGs could be detected in each sample.

Taxonomic richness (*s*) is either distributed as a Poisson random variable (variance ≈ mean), or as a negative binomial random variable if the variance in richness is greater than the mean. The latter would suggest that a few hosts carry a very diverse community of microbes relative to others. If the number of ARGs per microbial taxon is sensitive to the relative abundance of microbial species, and/or some species are more likely than others to carry ARGs, we would expect that the probability *p* of carrying an ARG would vary from sample to sample. This would suggest that *pN* is distributed as a beta-binomial random variable. Alternatively, if horizontal transfer of ARGs has a greater effect than the identity of microbial taxa, we would expect *pN* to be distributed as a binomial random variable. The combinations of these four possibilities define hypotheses for the distribution of ARGs in the community (see Table 2).

**Table 2:** Hypotheses for the distribution of ARGs in the community.

Pr[z] = Pr[x]∙Pr[y]	Genus Richness	RA Genes∙Taxon^-1^
ΣΣe−λλxx!.(ny)py(1−p)n−y	$\frac{mean}{var}\approx 1$	*p* constant
ΣΣr(k+x)r(k)x!∙(kK+μ)k∙(μK+μ)x∙(Ny)py(1−p)N−y	$\frac{mean}{var}>1$	*p* constant
$\Sigma \Sigma \frac{e^{-\lambda }\lambda^{x}}{x!}∙\frac{\Gamma (a+b)∙\Gamma (y+a)∙\Gamma (N-y+b)∙N!}{\Gamma (a)∙\Gamma (b)∙(N+a+b)∙(N-y)!∙y!}$	$\frac{mean}{var}\approx 1$	*P* variable
$\Sigma \Sigma \frac{\Gamma (\kappa +x)}{\Gamma (\kappa )x!}∙{\left(\frac{k}{k+\mu }\right)}^{k}∙{\left(\frac{\mu }{k+\mu }\right)}^{x}∙\frac{\Gamma (a+b)∙\Gamma (y+a)∙\Gamma (N-y+b)∙N!}{\Gamma (a)∙\Gamma (b)∙\Gamma (N+a+b)∙(N-y)!∙y!}$	$\frac{mean}{var}>1$	*p* variable

### Quantitative PCR ARG assay

DNA isolated from multiple anatomical locations, except the trabecular space and interhemispheric fissure, from twenty cases was combined to obtain samples representing an entire case, and screened for ARGs using the Qiagen 96-well Microbial DNA qPCR Array (S1 Table). This array detects 84 ARGs across multiple antibiotic classes including: aminoglycoside, beta-lactam, fluoroquinolone, glycopeptide, macrolide, tetracycline, and multidrug efflux pumps. Each assay plate contains two pan bacteria and one positive PCR controls to test for the presence of inhibitors and PCR efficiency. Along with the positive controls, two full plates of no template controls were used as negative control assays replacing sample template with nuclease-free water to ensure no cross contamination or false positives arose from our reagents or procedure. A full list of the genes detected in the assay can be found in the manufacture’s user manual. For each case, DNA was pooled from the eyes, ears, nares, mouth, umbilicus, and rectum in roughly equal concentrations to create a 500 ng pooled DNA sample. Four cases (HJ025, HJ027, HJ030, and HJ036) only contained 5 anatomical sites since DNA was not successfully extracted from one site (rectum, umbilicus, umbilicus, and mouth, respectively). A BioRad C1000 Touch Thermocycler with CFX96 Real-Time System was used to perform quantitative PCR using initial cycling conditions consisting of 10 minutes at 95°C, followed by 40 cycles of: 15 seconds at 95°C and 2 minutes 60°C with FAM fluorophore detection. Values for Cqs were recorded for each well and 20.0–37.99 was chosen as the range for positive detection as recommended by the assay manufacturer.

### Statistical information

Basic statistical analyses of antibiotic resistance gene counts were performed to determine the minimum, maximum, mean and standard deviation if the data was considered normal by a Shapiro-Wilks test and the minimum, maximum, mean, median, and interquartile ranges were determined if the data was considered non-normal using the basic stats package in the R statistical program v3.4.0[54].

Shannon diversity index (H’) and rank abundances were calculated for each case based on the genera taxonomic level relative abundance using the vegan package v2.4–4 in R[55]. A one-way ANOVA followed by Tukey’s honest significant difference test from the basic stats package of R was used to compare between H’ indices and each effect of the metadata (i.e. sex, age range, sample year, race, BMI, case discovery site, and manner of death)[54]. We removed effects that contained variables with fewer than three samples from the analysis since not enough data was present to perform parametrical statistics. A MANOVA followed by Tukey’s honest significance difference test was used to compare between H’ indices and each possible two effect pairing, but a model could not be created with more than two effects due to limitations in sample size. Recognizing that the inability to create one model that accounts for all the effects increases the chance for false positives, we performed a Bonferroni correction to obtain a new alpha value for the ANOVAs (α = 0.007) and the MANOVAS (α = 0.002). Statistical power analyses for one-way ANOVA was performed with the R pwr package v.1.2–1 to confirm that the number of samples were too small to detect large variation between the metadata populations[56]. Parameters included the single effect alpha value above, power value of 0.80, group number determined per metadata section, and effect size of 0.40 to detect large differences.

We estimated maximum likelihood estimates (MLEs) for the theoretical models using the mle2 function from the bbmle package in R and compared models using the AIC function to determine the best model[54, 57]. We also wanted to confirm that the detection of ARGs was not a function of the metagenome sizes (number of nucleotides) above the cut-off. This was tested by creating a linear regression model in R of both the square root transformed metagenome sizes (independent variable) and number of ARGs detected (dependent variable) (S1 and S3 Tables). The correlation significance was determined with Spearman’s rank correlation in R[54].

## Supporting information

S1 Table Metadata table of cases sampled during routine autopsy. Attributes that are not known are designated with “NA”. Starting from the first column, the case ID is the identification used for the study, the test that were performed on the case (S = sequenced, S* = sequenced but did not meet metagenome size threshold to be analyzed, Q = qPCR analysis), sampled locations (T = trabecular space, I = interhemispheric fissure, W = “whole case”), race (B = black, BH = black-hispanic, W = white), sex (M = male, F = female), age group (Y = youth [0-17yo], YA = young adult [18-25yo], A = adult [26-40yo], MA = middle aged adult [41-60yo], OA = old aged adult [61+yo]), BMI group (UN = underweight [<18.5], N = normal [18.5–24.9], OV = overweight [25–29.9], OB = obese [30+]), the case discovery site (I = indoors, O = outdoors, H = hospital), broad manner of death (N = natural, A = accident, H = homicide, S = suicide), and estimated postmortem interval.
(XLSX)

S2 Table Table of metagenome assemblies and analyses data. Represented are the details of the metagenome assemblies along with the information of each metagenome before the anatomical sites were combined for the community structure determination. The combined anatomical locations have the same case ID and share the same genera richness and H index as represented by the “#C” for the combined number. The different numbers of ARGs detected between the anatomical locations is also displayed. The anatomical location is represented as (TRA) for trabecular space of occipital bone and (INT) for interhemispheric fissure. The greyed out lines represent the metagenomes removed from analyses as discussed within the Materials and Methods.
(XLSX)

S3 Table Table of ARG counts across the study with CARD ARO accessions. (XLSX)
